# CUMULATIVE DAILY DISCRIMINATION AS A RISK FACTOR FOR REDUCED PURPOSE

**DOI:** 10.1093/geroni/igac059.478

**Published:** 2022-12-20

**Authors:** Megan Wilson, Lydia Ong, Gabrielle Pfund, Anthony Burrow, Nancy Sin

**Affiliations:** Washington University in St. Louis, St. Louis, Missouri, United States; University of British Columbia, Vancouver, British Columbia, Canada; Northwestern University, Chicago, Illinois, United States; Cornell University, Ithaca, New York, United States; University of British Columbia, Vancouver, British Columbia, Canada

## Abstract

Past research shows that discriminatory experiences may reduce sense of purpose among older adults, though these associations are inconsistent across groups. Work is needed both to understand the nuanced role discrimination plays on individuals’ sense of purpose, whether it leads to feeling derailed from life goals, and if effects differ for younger versus older adults. The current study asked 354 American adults (age 19-74) to complete daily surveys over the course of two weeks, to examine whether experiencing discrimination during that two-week period led to a decline in purposefulness. Overall, findings suggest marked stability in sense of purpose during this short timeframe. However, greater daily discrimination predicted decreases in sense of purpose, and increases in derailment over the two weeks. While these associations were similar across age, older adults did report less discrimination at baseline. Findings will be discussed with a focus on successful aging among marginalized groups.

